# Relations between Automatically Extracted Motion Features and the Quality of Mother-Infant Interactions at 4 and 13 Months

**DOI:** 10.3389/fpsyg.2017.02178

**Published:** 2017-12-13

**Authors:** Ida Egmose, Giovanna Varni, Katharina Cordes, Johanne Smith-Nielsen, Mette S. Væver, Simo Køppe, David Cohen, Mohamed Chetouani

**Affiliations:** ^1^Early Child Developmental Unit, Babylab, Department of Psychology, University of Copenhagen, Copenhagen, Denmark; ^2^LTCI, Télécom ParisTech, Université Paris-Saclay, Paris, France; ^3^Department of Child and Adolescent Psychiatry, Pitié-Salpêtrière Hospital, Sorbonne Université Pierre et Marie Curie, Paris, France; ^4^Institut des Systèmes Intelligents et de Robotiques, Centre National de la Recherche 7222, Sorbonne Université Pierre et Marie Curie, Paris, France

**Keywords:** movement, motion features, mother-infant interaction, interaction quality, coding interactive behavior

## Abstract

Bodily movements are an essential component of social interactions. However, the role of movement in early mother-infant interaction has received little attention in the research literature. The aim of the present study was to investigate the relationship between automatically extracted motion features and interaction quality in mother-infant interactions at 4 and 13 months. The sample consisted of 19 mother-infant dyads at 4 months and 33 mother-infant dyads at 13 months. The coding system Coding Interactive Behavior (CIB) was used for rating the quality of the interactions. Kinetic energy of upper-body, arms and head motion was calculated and used as segmentation in order to extract coarse- and fine-grained motion features. Spearman correlations were conducted between the composites derived from the CIB and the coarse- and fine-grained motion features. At both 4 and 13 months, longer durations of maternal arm motion and infant upper-body motion were associated with more aversive interactions, i.e., more parent-led interactions and more infant negativity. Further, at 4 months, the amount of motion silence was related to more adaptive interactions, i.e., more sensitive and child-led interactions. Analyses of the fine-grained motion features showed that if the mother coordinates her head movements with her infant's head movements, the interaction is rated as more adaptive in terms of less infant negativity and less dyadic negative states. We found more and stronger correlations between the motion features and the interaction qualities at 4 compared to 13 months. These results highlight that motion features are related to the quality of mother-infant interactions. Factors such as infant age and interaction set-up are likely to modify the meaning and importance of different motion features.

## Introduction

Bodily movements are an essential component of social interactions throughout life (Argyle, 1988). The way we move, and the way we coordinate our movements with our interaction partner, convey information on, for instance, relationship quality (Ramseyer and Tschacher, 2011, 2014), emotional states (for a review, see Kleinsmith and Bianchi-Berthouze, 2013), and personality traits (e.g., Anzalone et al., 2017). In early parent-infant interactions, bodily movements constitute a central part of the stimulation alongside other modalities, such as vocalizations, gaze, and facial affect (Beebe, 2010).

Well-balanced parent-infant interactions include both periods of engagement and of disengagement (Stern, 1974; Bowlby, 1988; Væver et al., 2010; Guedeney et al., 2013; Beebe, 2017). Parents need to stimulate their infants in order to engage them, but they also need to accept the infant's signs of disengagement and social withdrawal (Guedeney et al., 2013), such as gaze aversion and head turning, by reducing the level of stimulation. During pauses, the infant gains time to regulate his or her level of arousal or to take initiatives in the interaction (Beebe, 2010). Thus, pauses and disengagement are as central a part of early parent-infant interaction as periods of stimulation and engagement. The optimal amount of stimulation in early parent-infant interactions is described by the “mid-range model”; both too high and too low levels of parental stimulation are related to insecure child development (Beebe and Steele, 2013).

Research on parent-infant interactions has mainly focused on communication through vocalizations, facial expressions, and gaze behaviors (Pérez and Español, 2016). Thus, apart from pioneering studies describing patterns of coordination between infant and maternal movements (Stern, 1971; Beebe et al., 1977, 2010; Beebe, 1982), little is known on the role of movement communication in early parent-infant interaction.

One reason for the lack of research on bodily movements may be the difficulty and extremely time-consuming process of segmenting and annotating such movements manually. However, the past decades' advances in motion-tracking computer systems offer still more accurate and less time-consuming ways of capturing, extracting and analyzing motion features in interactions. While these techniques have been used in studies on motor development for many years (e.g., von Hofsten and Rönnqvist, 1988), their use in adult-infant interactions is relatively new. However, a few studies have applied motion capture systems on adult-infant interactions. For example, Væver et al. (2013) reported that the variability in head distance between mothers and infants was lower for mothers with postpartum depression than for non-clinical mothers. In another study, Leclère et al. (2016) showed that it was possible to discriminate between non-clinical mothers and mothers who were emotionally neglecting, based on motion features such as motion activity, pause, and overlapping motion. Finally, Delaherche et al. (2013) demonstrated that it was possible to discriminate between autistic and non-clinical children interacting with therapists using motion features such as gestural and pause durations. Although the studies are few, their results suggest that specific types of motion features and movement coordination may be associated with specific types of psychological difficulties and may be specific for clinical groups.

Other studies have linked certain motion features and patterns of coordination to the quality of the interaction. Using a placebo crossover experimental design, Weisman et al. (2012, 2013) showed that oxytocin was associated with better social reciprocity during free play interaction, and that oxytocin-modulated parental proximity to the infant, as well as fathers' head speed and acceleration. It could be that the oxytocin-induced alterations in fathers' motion characteristics increased the level of motionese, i.e., moving in an exaggerated way, which enhanced the infant's attention. Leclère et al. (2016) found that higher levels of motion activity, overlapping motion, and contingent motion responses in mother-infant interactions were associated with higher levels of aversive interaction qualities, such as maternal intrusiveness and child avoidance, and with lower levels of adaptive interaction qualities, such as maternal sensitivity. In line with these results, other studies have found that an increase in the velocity of infant head movements is related to an increase in infant negative affect and distress within mother-infant interactions at 4 and 13 months of infant age (Hammal et al., 2015a,b).

The relationship between motion features and interaction quality has also been investigated in adult-adult interactions. Hammal et al. (2014) demonstrated that, in interactions between romantic partners, periods with conflict were characterized by increased levels of head motion, but decreased levels of coordination. Within the psychotherapeutic context, Ramseyer and Tschacher (2011) showed that higher levels of in-session motion synchrony were associated with higher patient ratings of the relationship quality after the session and higher levels of symptom reduction upon termination of the therapy. Furthermore, Ramseyer and Tschacher (2014) differentiated between head- and upper-body-synchrony, and showed that head-synchrony predicted the global treatment outcome, while upper-body synchrony predicted the session outcome.

Taken together, the studies on movement in adult-adult and infant-adult interaction indicate that high levels of motion activity are related to more aversive interactions, e.g., periods with interpersonal conflict or intra-personal distress. In fact, Hammal et al. (2015a) suggest a continuum with increasing levels of motion activity going from depressed, to neutral, to positive and finally negative affect. The previous studies, excepting the results from Leclère et al. (2016), also suggest that higher levels of movement coordination, i.e., where the partners adjust their level of motion activity to each other, are associated with non-conflictual periods and a better rating of the relationship. In mother-infant interaction, when the mother coordinates her movements to her infant's, she shows the infant that she is aware of him and that she would like to participate in his experience (Stern, 2004).

### The present study

The objective of the present study was to examine the relationships between upper-body, arm, and head movement and interaction quality in face-to-face interactions between normal, i.e., non-clinical, mothers and their infants at 4 and 13 months of age. The study aims at contributing to the growing area of research on movement in early parent-infant interactions by showing how different types of global interaction qualities, such as maternal intrusiveness or dyadic reciprocity, can be described in terms of movement at different infant ages. Thus, the study examines whether some movement patterns, e.g., high levels of maternal movement or low levels of motion silence, are consistently related to adaptive or aversive interactions.

Infant ages 4 and 13 months were chosen, as there are important changes in interactional quality at these ages, due to the infant's social development (Rochat and Striano, 1999). At 4 months, parent-infant interactions are characterized by primary intersubjectivity, that is parent and infant engage in reciprocal face-to-face interactions coordinating their attention within the dyad. At 13 months, the secondary intersubjectivity is developed, and at this time, parent-infant interactions are also characterized by coordination of attention on objects in the environment (Trevarthen, 1998; Rochat and Striano, 1999).

The study is based on the results from the study by Leclère et al. (2016) who examined the role of upper-body motion in a pre-determined play situation between 12- and 36-month-old children and emotionally neglecting or non-clinical mothers. The present study extends the results from the study by Leclère et al. (2016) by investigating whether the same relationships between motion features and interaction qualities are present for different body parts, i.e., arm, head, and upper-body motion, at different infant ages, specifically, 4 and 13 months, and in a different interaction arrangement, namely a free face-to-face interaction.

In the present study, the interaction quality is evaluated using the global rating system Coding Interactive Behavior (CIB; Feldman, 1998, 2012; Keren et al., 2001). According to the CIB, adaptive and positive interactions are characterized as child-led, i.e., focused on the infant's needs and states, and they include high levels of maternal sensitivity, maternal limit-setting, infant involvement, infant compliance, and dyadic reciprocity. On the other hand, adverse and negative interactions are characterized as parent-led, i.e., focused on the parent's needs and plan of action, and include high levels of maternal intrusiveness, infant negativity, and dyadic negative states (Feldman, 2012; Leclère et al., 2014).

Based on previous findings, we expect (a) *infant and mother motion activity* plus *overlap*, i.e., periods where mother and infant move simultaneous, to be positively correlated with aversive interaction qualities and negatively correlated with adaptive interaction qualities, (b) *motion silence*, i.e., periods where neither child nor mother move, to be positively correlated with adaptive interaction qualities and negatively correlated with aversive interaction qualities, and (c) *motion coordination* to be positively correlated with adaptive interaction qualities and negatively correlated with aversive interaction qualities. Furthermore, inspired by Ramseyer and Tschacher's (2014) results showing that head- and upper-body-synchrony are differently related to therapeutic outcomes, we explore whether motion features derived from the mothers' and infants' heads and arms, considered separately, are related differently to interaction qualities in mother-infant interactions.

## Materials and methods

### Participants

The participants involved in the current study were enrolled in a larger longitudinal study investigating early mother-infant interactions conducted at the University of Copenhagen Babylab. The study was approved by The Research Ethics Committee, Department of Psychology, University of Copenhagen. All mothers gave written and informed consent prior to participation.

**Figure 1:**
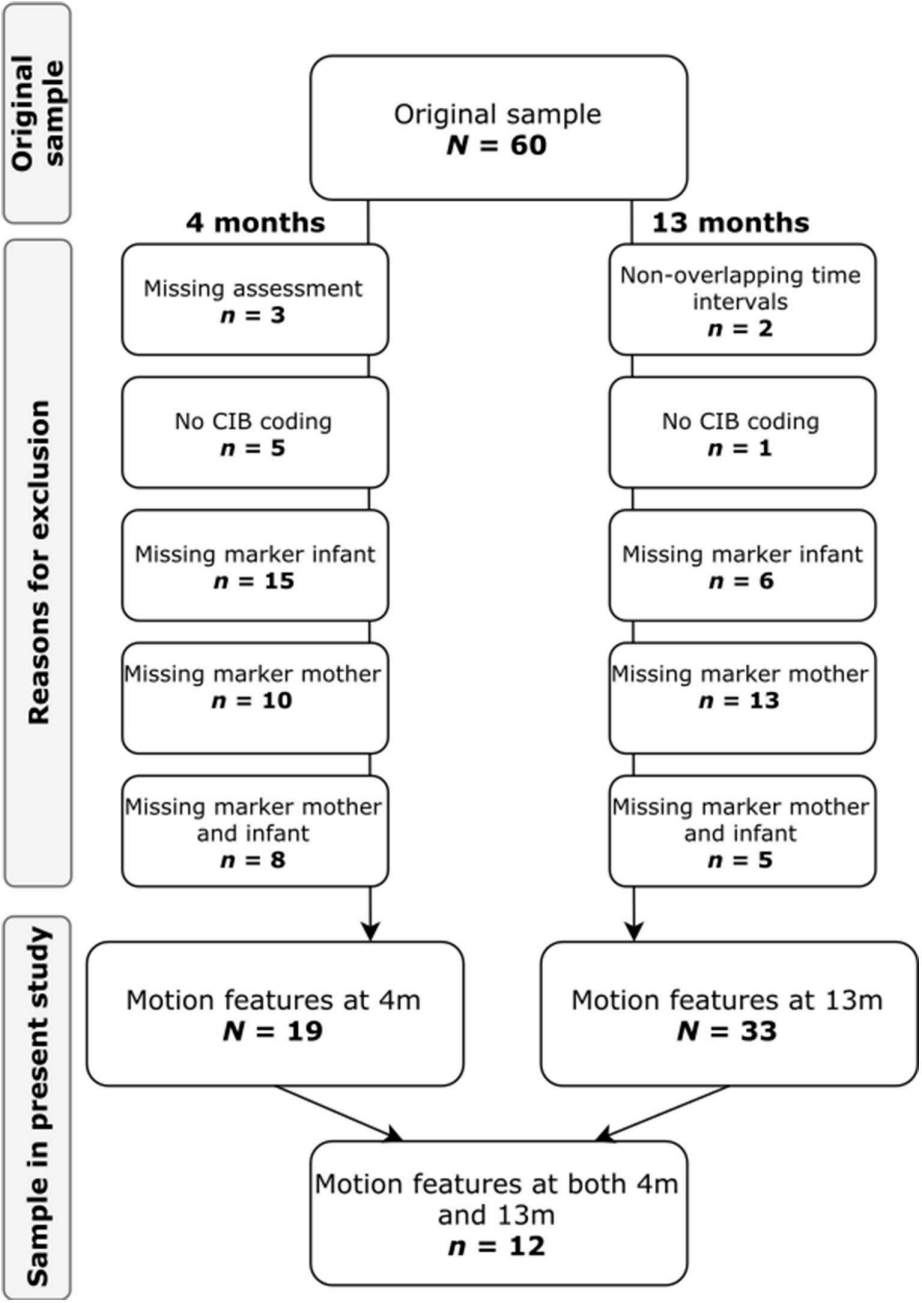
Flow of participants. The figure displays the flow of participants from the original sample to the present study at 4 and 13 months, respectively.

The original sample consisted of 60 non-clinical mother-infant dyads followed from pregnancy to 13 months. The present study examined 19 mother-infant dyads at 4 months (10 girls; 9 boys) and 33 mother-infant dyads at 13 months (18 girls; 15 boys). Of the 19 mother-infant dyads included in the 4-months-sample, 12 were also included in the 13-month-sample. Mother-infant dyads were excluded due to missing assessments and technical reasons, such as video being non-codable for CIB due to e.g., maternal facial expressions not being visible on the video recording or missing marker data for either mother and/or infant due to e.g., visual occlusion of the markers. Figure 1 displays a detailed flowchart of how the dyads analyzed in the present study were selected.

Recruitment of the mothers proceeded as follows. The participants contacted the research unit in response to advertisements on maternity-related web pages and at local obstetricians which invited volunteer participants for a longitudinal study on mother-infant interaction. Mothers were included if they were pregnant with a singleton pregnancy, primiparous, and somatically and psychologically well. Criteria for exclusion were severe neurological or somatic disorder in the mother within 1 year postpartum, premature birth or major physical or mental disabilities in the child after birth.

### Procedure

Mother-infant interactions were recorded using video cameras and a motion capture system in visually neutral and sound proof observation room. The video recordings were used to analyse the quality of the interactions using the CIB, while the motion capture recordings were used to calculate the kinetic energy of upper-body, arms, and head motion. The recordings were timed to fit the infants' sleeping and eating patterns. Each interaction lasted 10 min. The mother and the infant were seated in a standard face-to-face setup with the infant seated in an infant-seat in front of the mother (Tronick et al., 1989). At 4 months, the infants were seated in a chair supporting the back, which was slightly tilted and fixed in an upright position without being uncomfortable for the infant. At 13 months, the infants were able to sit self-supported, and were seated in a high chair with more freedom to move. Two video cameras (Panasonic NV-GS300, PAL; 25 fps), one placed behind the mother and one placed next to the infant and the mother, recorded the frontal view of the infant and the lateral view of the dyad, respectively. Before the beginning of the interaction, the mothers were instructed to be with their infants as they normally would.

**Figure 2:**
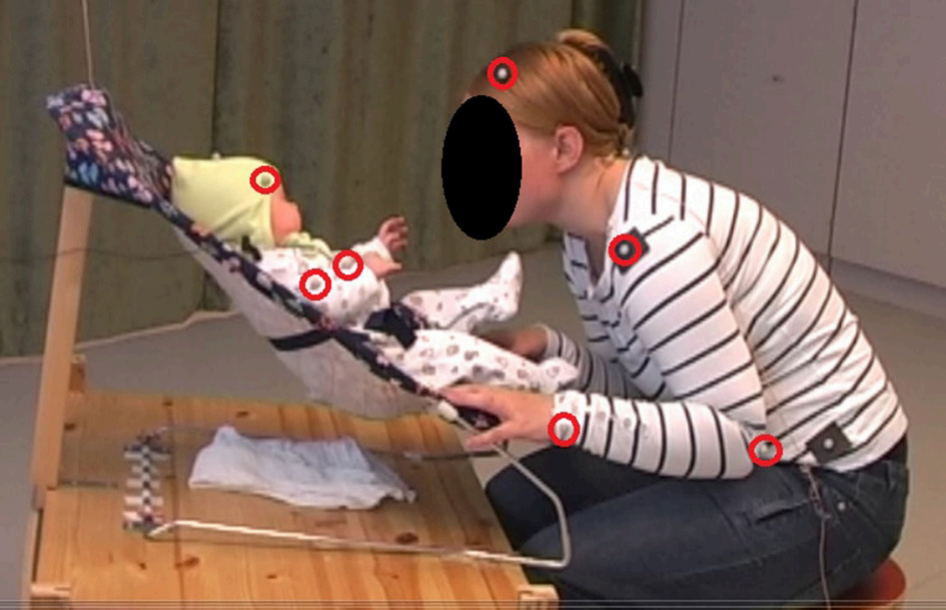
Marker placement at 4 months. The image displays the interaction set-up at 4 months The makers used in the calculation of motion features are marked with red. The calculations include the corresponding markers on the opposite body side.

**Figure 3:**
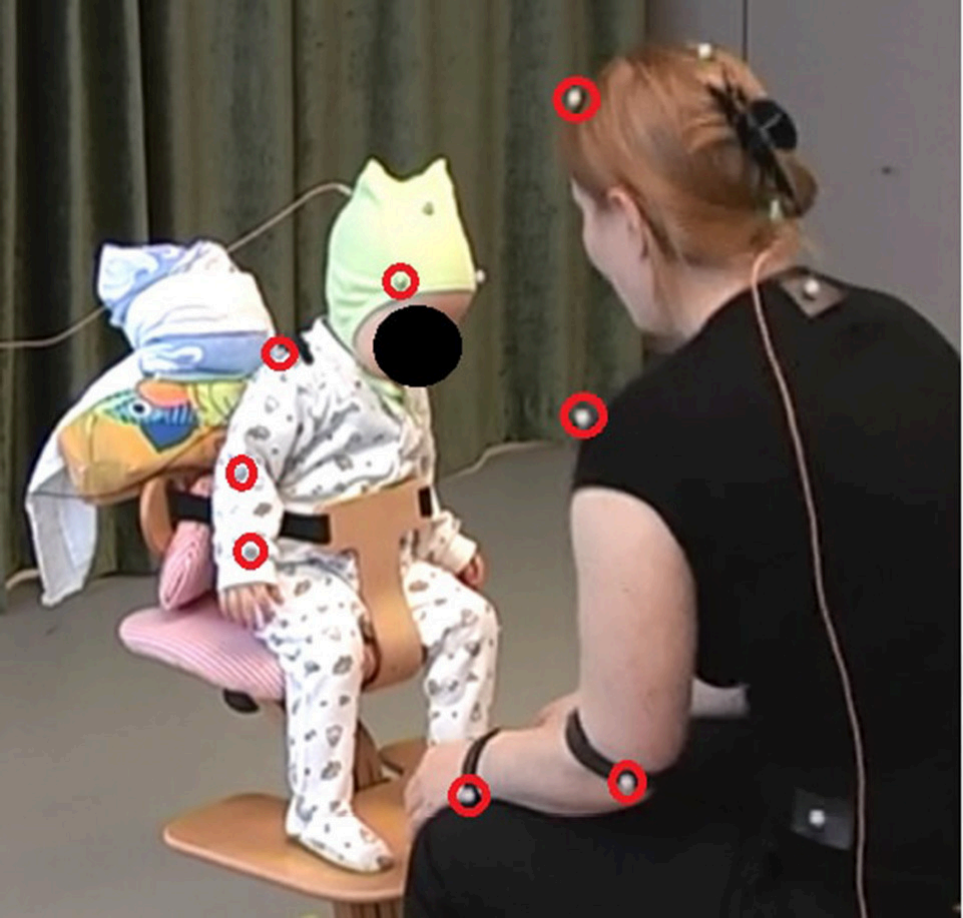
Marker placement at 13 months. The image displays the interaction set-up at 13 months The makers used in the calculation of motion features are marked with red. The calculations include the corresponding markers on the opposite body side.

The motion capture system was an 8-camera optoelectronic registration system using spherical infra-red passive reflective markers (diameter = 12 mm) (ProReflex, 240 Hz; Qualisys Inc., Gotenburg, Sweden^1^. For the mothers, the markers were placed on Velcro straps. For the infants, the markers were stuck onto a hat and body stocking. The markers used in the present study were attached to the head, wrists, elbows, and shoulders on each side of the body on the mother and the infant, respectively. However, due to missing data and limited movability of the shoulders, the shoulder markers were not used in the calculations of the infants' kinetic energy at 4 months (see Figures 2, 3).

### Global rating of the quality of the interaction

The interaction quality of the mother-infant interactions was assessed using the CIB (Feldman, 1998). The CIB consists of 22 parent scales, 16 infant scales, five dyadic scales, and two scales expressing the lead-lag in the interaction, and can be used for rating interactions between adults and infants aged 2–36 months. The scales are rated on a 5-point Likert scale with 1 indicating a minimal level of a specific behavior or attitude and 5 indicating a maximal level.

In order to ensure that each dyad had time to accustom to the interactive set-up in the experimental room, video recordings were rated from 2 min and onwards. The 4-month-interactions were rated for three consecutive minutes; the 13-months-interactions were rated for five consecutive minutes. The 4-month-interactions were rated by the co-authors KC and JSN, who have been trained by the developer of the CIB (Feldman, 1998). The 13-month-interactions were rated by two coders trained by KC and JSN. In order to ensure consistency in the ratings at 4 and 13 months, the raters at 13 months had attained an average percentage agreement above 80% on 12 videos with the raters at 4 months before rating the 13-month-interactions. A randomly selected subset (20%) of the videos at 4 and 13 months were double-coded for interrater-reliability (Bakeman and Quera, 2011). Inter-rater reliability was calculated at the scale level using Intra-Class Correlations (ICC) and their 95% confidence intervals (CI) based on a single measures, absolute agreement, two-way random effects model (Koo and Li, 2016). The scales showed good to excellent reliability, as indicated by ICC_4m_(2,1) = 0.89, 95% CI 0.87–0.91; ICC_13m_(2,1) = 0.89, 95% CI 0.88–0.91.

Sub-scales were averaged into theoretically meaningful composites proposed by Ruth Feldman and they showed acceptable to high levels of internal consistency (Cronbach's alpha = 0.75–0.94) (Keren et al., 2001; Feldman, 2012). See Table 1 for an overview of the CIB composites calculated, the scales included in the composites, and the internal consistency for each composite. In addition to the CIB composites, we also included the two scales expressing the lead-lag in the interaction: *Child-led* expressing the degree to which the interaction was focused on the infant's needs and states, and *Parent-led* expressing the degree to which the interaction was focused on the parent's needs and plan of action. These scales were added, as they more broadly express, who the interaction is centered around and who's initiatives is followed in the interaction.

**Table 1:** CIB composites.

**CIB composite**	**Infant age**	**Scales included in composite**	**Cronbach's α**
Maternal sensitivity	4 and 13 m	Acknowledgement, Imitation (applies only to 4 m), Elaborating, Positive affect, Vocal appropriateness, Appropriate range of affect, Resourcefulness, Supportive presence	4 m = 0.86 13 m = 0.91
Maternal intrusiveness	4 and 13 m	Overriding	–
Maternal limit-setting	13 m	Consistency of style, On-task persistence, Appropriate structure	13 m = 0.89
Infant involvement	4 and 13 m	Gaze/Joint attention, Positive affect (only at 4 m), Alert, Vocalizations/Verbal output, Initiation, Fatigue (reversed) (only at 13 m), Competent use of environment (applies only to 13 m), Creative symbolic play (applies only to 13 m)	4 m = 0.84 13 m = 0.86
Infant negativity	4 and 13 m	Negative emotionality, Labile affect (applies only to 13 m)	4–13 m = 0.75
Infant compliance	13 m	Compliance to parent, On-task persistence	13 m = 0.79
Dyadic reciprocity	4 and 13 m	Dyadic reciprocity, Fluency, Adaptation-Regulation	4 m = 0.89 13 m = 0.94
Dyadic negative states	4 and 13 m	Constriction	–

*Overview of the CIB composites displaying the ages for which the composites are present, the scales included in the composites, and the internal consistency measured as the Cronbach's α for each composite at each infant age*.

### Automated extraction of motion features

The motion capture data were pre-processed as follows using the MoCap MATLAB Toolbox (Burger and Toiviainen, 2013). First, the motion capture recording from each mother-infant interaction was trimmed according to the time interval specified by the CIB (3 min for the 4-month-interactions and 5 min for the 13-month-interactions). Second, a linear interpolation was adopted to handle missing data. Such interpolation was applied only when the duration of missing data segments was less than 5% of the total duration of the interaction (i.e., 3 min for 4 months and 5 min for 13 months), otherwise the corresponding interaction was discarded (see Figure 1). The average percentage of time in which one or more markers were missing during the interaction segments was 2.7% for the mothers and 15.5% for the infants in the 4-month-interactions, and 7.8% for the mothers and 9.4% for the infants in the 13-month-interactions. Finally, data were low-pass filtered using a 4th order Butterworth filter with a cut-off frequency of 10 Hz. This value was chosen according to the literature on motion capture data processing (Skogstad et al., 2013). The translational kinetic energy, i.e., the energy related to movement, of the head (*K*_*h*_), the arms (*K*_*a*_) and of upper-body (*K*_*ub*_) of the mother and the infant was computed using an ad hoc EyesWeb XMI application (Piana et al., 2013). The values of the body segments' masses were expressed as fractions of the mass of the total body. The values of the mothers' masses were from the work of Winter (2009), which provides information from the most recognized anthropometric research studies conducted by the U.S. Air force. The values of the children's masses were not directly available from Winter (2009), and were conveniently rescaled from the masses of the mothers taking into account additional anthropometric studies carried out on children. The kinetic energy was calculated according the following formulas:

$$\begin{array}{lll} K_{h} & = & \frac{1}{2}m_{h}v_{h}^{2}\\ K_{a} & = & \;\frac{1}{2}m_{fa}v_{fa}^{2}\left(+\;\frac{1}{2}\;m_{ua}v_{ua}^{2}\right)2\\ K_{ub} & = & \frac{1}{2}m_{h}v_{h}^{2}+\frac{1}{2}m_{fa}v_{fa}^{2}\left(+\frac{1}{2}m_{ua}v_{ua}^{2}+\frac{1}{2}m_{s}v_{s}^{2}\right)3, \end{array}$$

where *m*_*h*_, *m*_*fa*_, *m*_*ua*_, *m*_*s*_, *v*_*h*_, *v*_*fa*_, *v*_*ua*_, *v*_*s*_ are the masses and the speeds of head, forearms and hands, upper arms, and shoulders, respectively. The values of speed refer to the center of mass of each segment.

The coarse- and fine-grained motion features used in the present study were computed from the translational kinetic energy as follows. A threshold-based segmentation of the kinetic energy was performed to identify segments of movement and no movement. More specifically, the average kinetic energy was computed for all mothers and infants by removing the sparse spikes occurring in the data due to impulsive movements. This was done in order to avoid obtaining biased values of the averages and did not affect segmentation, since the spikes by definition were higher than the threshold. The threshold value adopted was empirically fixed to 20% of the average kinetic energy for mothers and infants, respectively. Additionally, movements separated by pauses shorter than 350 ms were merged in a single movement, and movements shorter than 350 ms were discarded. This was done as the motion capture systems are able to detect pauses and movements of very short durations, which would not be likely to be considered as such by a human observer. The limit of 350 ms was chosen as this has been shown to be the mean reaction time to visual stimuli (Shelton and Kumar, 2010).

Following Leclère et al. (2016) and Varni et al. (2015), the below listed coarse-grained, global motion features were extracted:

- *Infant activity ratio*: the percentage of time in which the child was moving.
- *Maternal activity ratio*: the percentage of time in which the mother was moving.
- *Overlap ratio*: the percentage of time in which mother and child were moving simultaneously.
- *Silence ratio*: the percentage of time in which neither mother nor child were moving.

**Figure 4:**
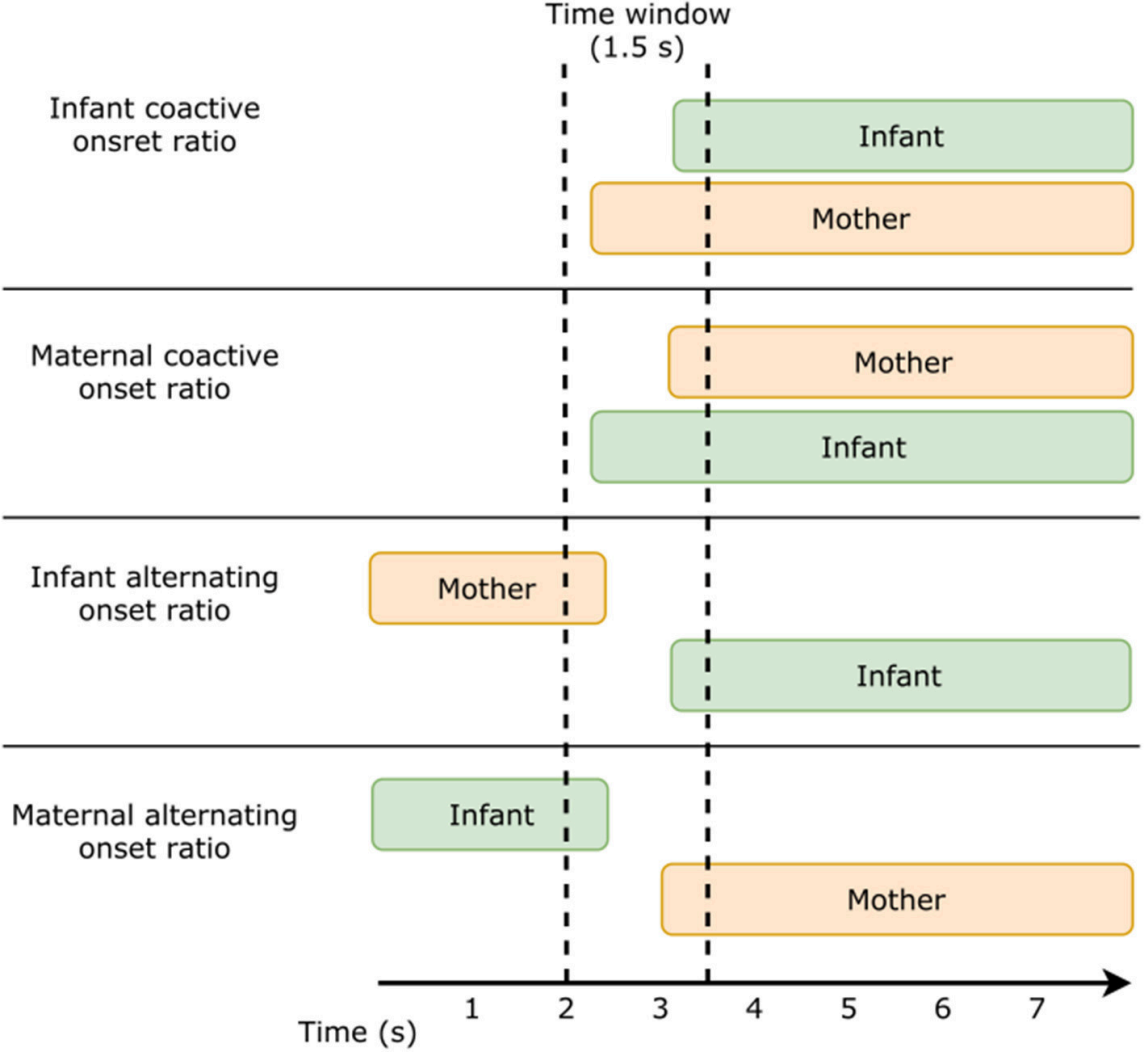
Fine-grained motion features. Schematic overview displaying the coordination of maternal and infant movement during infant coactive onset, maternal coactive onset, infant alternating onset, and maternal alternating onset.

The global motion features can be classified as either individual or dyadic. The infant and mother activity ratios are individual motion features, as they only are defined by the state of one partner, whereas the overlap and silence ratios are dyadic as they are defined by the state of both partners. Global motion features provide an overall impression of the interaction and coordination, i.e., are mother and child often moving simultaneously or not, but they do not provide information on the more fine-grained levels of coordination, such as synchrony and contingency, which previous studies have found to be important for the interaction quality and infant development (for a review, see Leclère et al., 2014). Thus, to assess the coordination of movement at a more fine-grained level of analysis, we extracted the following motion features (see Figure 4):

- *Infant coactive onset ratio*: the percentage of the infant's movements which occur (a) while the mother is moving and (b) within maximum 1.5 s after maternal movement onset^4^.
- *Maternal coactive onset ratio*: the percentage of the mother's movements which occur (a) while the infant is moving and (b) within maximum 1.5 s after infant movement onset.
- *Infant alternating onset ratio*: the percentage of the infant's movements which occur (a) while the mother is not moving, and (b) within maximum 1.5 s after maternal movement offset.
- *Maternal alternating onset ratio*: the percentage of the mother's movements which occur (a) while the infant is not moving, and (b) within maximum 1.5 s after infant movement offset.

These fine-grained motion features are dyadic, as they are defined by the movements of both the mother and the infant. The fine-grained motion features included in the present study differ from those included in previous studies (Delaherche et al., 2013; Leclère et al., 2016), as they are not only defined by the time-lag between partner B's movement onset or offset and partner A's movement onset, but also take into account, whether partner B is moving or not, when partner A starts moving. This differentiation may be important with regard to the function of the coordination. The alternating onset ratio resembles speech turn-taking (Holler et al., 2016), i.e., a type of coordination where the interaction partners act in alternating turns; initiating during periods of silence and timing the initiation to the other partner's offset. On the other hand, the coactive onset ratio expresses a simultaneous activity, where one partner's movement onset triggers the movement onset of the other partner, e.g., an act of imitation, where the infant starts clapping, whereupon this activity engages the mother to clap with the infant.

**Table 2:** Spearman's correlations between global motion features and CIB composites for mother-infant dyads at 4 months (*N* = 19).

**4 m**	**Global motion features**	**Body part**	**CIB Composites**							
			**Maternal sensitivity**	**Maternal intrusiveness**	**Infant involvement**	**Infant negative emotionality**	**Dyadic reciprocity**	**Dyadic negative states**	**Parent–led**	**Child–led**
Individual parameters	Infant activity ratio	Upper–body	−0.61 *p* = 0.01	0.39 *p* = 0.10	−0.31 *p* = 0.20	0.46 *p* = 0.05	−0.32 *p* = 0.18	0.07 *p* = 0.79	0.13 *p* = 0.59	−0.38 *p* = 0.11
		Arms	−0.54 *p* = 0.02	0.43 *p* = 0.07	−0.29 *p* = 0.23	0.61 *p* = 0.01	−0.31 *p* = 0.20	0.29 *p* = 0.23	0.06 *p* = 0.79	−0.13*p* = 0.60
		Head	−0.59 *p* = 0.01	0.41 *p* = 0.08	−0.42 *p* = 0.07	0.44 *p* = 0.06	−0.36 *p* = 0.14	0.04 *p* = 0.86	0.22 *p* = 0.37	−0.45*p* = 0.051
	Maternal activity ratio	Upper–body	−0.07 *p* = 0.78	0.54 *p* = 0.02	−0.15 *p* = .55	0.12 *p* = 0.63	−0.14 *p* = 0.56	0.09 *p* = 0.72	0.49 *p* = 0.03	−0.16 *p* = 0.51
		Arms	−0.26 *p* = 0.29	0.62 *p* = 0.01	–.35 *p* = .15	0.19 *p* = 0.44	−0.34 *p* = 0.16	0.29 *p* = 0.22	0.66 *p* = 0.002	−0.19*p* = 0.44
		Head	0.14 *p* = 0.56	0.18 *p* = 0.45	.16 *p* = 0.53	0.13 *p* = 0.60	0.22 *p* = 0.37	−0.31 *p* = 0.20	0.01 *p* = 0.95	−0.07*p* = 0.77
Dyadic parameters	Overlap ratio	Upper–body	−0.39 *p* = 0.10	0.48 *p* = 0.04	−0.29 *p* = 0.23	0.46 *p* = 0.05	−0.21 *p* = 0.38	0.13 *p* = 0.60	0.25 *p* = 0.31	−0.18*p* = 0.47
		Arms	−0.44 *p* = 0.06	0.50 *p* = 0.03	−0.35 *p* = 0.14	0.52 *p* = 0.02	−0.30 *p* = 0.21	0.33 *p* = 0.17	0.33 *p* = 0.17	−0.09*p* = 0.72
		Head	−0.29 *p* = 0.23	0.36 *p* = 0.13	−0.14 *p* = 0.58	0.28 *p* = 0.24	−0.06 *p* = 0.81	−0.12 *p* = 0.61	0.16 *p* = 0.52	−0.27*p* = 0.27
	Silence ratio	Upper–body	0.56 *p* = 0.01	−0.55 *p* = 0.01	0.34 *p* = 0.16	−0.34 *p* = 0.15	0.42 *p* = 0.08	−0.12 *p* = 0.64	−0.41 *p* = 0.08	0.53*p* = 0.02
		Arms	0.55 *p* = 0.02	−0.62 *p* = 0.01	0.45 *p* = 0.054	–0.46 *p* = 0.05	0.52 *p* = 0.02	−0.40 *p* = 0.09	−0.48 *p* = 0.04	0.31*p* = 0.19
		Head	0.37 *p* = 0.13	−0.37 *p* = 0.12	0.12 *p* = 0.63	−0.40 *p* = 0.09	0.14 *p* = 0.56	0.24 *p* = 0.33	−0.14 *p* = 0.58	0.46 *p* = 0.05

*Color indicates that the correlation is statistically significant at the 0.05 level (2-tailed)*.

### Statistical analyses

The data were analyzed using IBM® SPSS® Statistics 24.0 (IBM Corp. Released 2016. IBM SPSS Statistics for Windows, Version 24.0. Armonk, NY: IBM Corp.), with two-tailed tests, and a 5% α-level. Since the CIB composite scores were not normally distributed, Spearman's correlation coefficients were used for assessing the relations between CIB composites and motion features at 4 months and 13 months.

## Results

All correlations between motion features and CIB composites are displayed in Tables 2–**5**.

### Relations between global motion activity and interaction quality

First, we hypothesized infant and maternal motion activity plus overlap to be positively correlated with aversive interaction qualities and negatively correlated with adaptive interaction qualities. As shown in Tables 2, 3, our findings primarily support the first part of the hypothesis. First, the infant activity ratios are positively correlated with infant negativity at both 4 and 13 months; at 4 months infant negativity is correlated with the activity ratios for upper-body and arm motion, while it is correlated with the activity ratios for upper-body and head motion at 13 months. Second, the maternal activity ratios are positively correlated with parent-led at both 4 and 13 months; at 4 months parent-led is correlated with the activity ratios for upper-body and arm motion, while it is correlated with the activity ratio for arm motion at 13 months. Further, the maternal activity ratios for upper-body and arm motion are positively correlated with maternal intrusiveness at 4 months. Third, the overlap ratios are positively correlated with infant negativity at both 4 and 13 months; at 4 months infant negativity is correlated with the overlap ratios for upper-body, arm and head motion, while it is correlated with the overlap ratios for upper-body and arm motion at 13 months. Further, the overlap ratios for upper-body and arm motion are positively correlated with maternal intrusiveness at 4 months. In addition to this, supporting the last part of the hypothesis, the infant activity ratios for upper-body, arm, and head motion were negatively correlated with maternal sensitivity at 4 months, while the maternal activity ratio for arm motion was negatively correlated with child-led at 13 months.

**Table 3:** Spearman's correlations between fine-grained motion features and CIB composites for mother-infant dyads at 4 months (*N* = 19).

**4 m**	**Fine–grained motion features**	**Body part**	**CIB Composites**							
			**Maternal sensitivity**	**Maternal intrusiveness**	**Infant Involvement**	**Infant negative emotionality**	**Dyadic reciprocity**	**Dyadic negative states**	**Parent–led**	**Child–led**
Dyadic parameters	Infant alternating onset ratio	Upper-body	0.15 *p* = 0.55	−0.01 *p* = 0.96	−0.12 *p* = 0.64	0.21 *p* = 0.39	0.17 *p* = 0.49	0.04 *p* = 0.88	−0.13 *p* = 0.61	0.47 *p* = 0.04
		Arms	−0.11 *p* = 0.66	0.05 *p* = 0.83	0.06 *p* = 0.80	0.57 *p* = 0.01	0.02 *p* = 0.94	0.13 *p* = 0.60	−0.10 *p* = 0.69	0.14 *p* = 0.58
		Head	0.34 *p* = 0.16	0.27 *p* = 0.26	0.17 *p* = 0.48	0.27 *p* = 0.27	0.37 *p* = 0.12	−0.19 *p* = 0.43	−0.10 *p* = 0.69	0.46 *p* = 0.05
	Maternal alternating onset ratio	Upper-body	0.46 *p* = 0.05	−0.30 *p* = 0.22	0.52 *p* = 0.02	−0.42 *p* = 0.07	0.31 *p* = 0.20	−0.27 *p* = 0.27	−0.17 *p* = 0.49	0.34 *p* = 0.16
		Arms	−0.02 *p* = 0.95	−0.24 *p* = 0.33	0.13 *p* = 0.59	−0.04 *p* = 0.86	0.05 *p* = 0.85	−0.03 *p* = 0.92	−0.05 *p* = 0.85	0.04 *p* = 0.87
		Head	0.54 *p* = 0.02	−0.47 *p* = 0.04	0.80 *p* < 0.001	−0.36 *p* = 0.13	0.70 *p* = 0.001	−0.55 *p* = 0.01	−0.57 *p* = 0.01	0.36 *p* = 0.13
	Infant coactive onset ratio	Upper-body	0.41 *p* = 0.08	−0.14 *p* = 0.56	0.37 *p* = 0.12	−0.04 *p* = 0.86	0.23 *p* = 0.35	−0.26 *p* = 0.29	−0.24 *p* = 0.33	0.37 *p* = 0.12
		Arms	0.01 *p* = 0.97	0.07 *p* = 0.78	−0.07 *p* = 0.78	−0.02 *p* = 0.94	0.01 *p* = 0.97	0.05 *p* = 0.83	0.33 *p* = 0.17	0.10*p* = 0.68
		Head	0.05 *p* = 0.85	−0.03 *p* = 0.90	0.38 *p* = 0.11	0.14 *p* = 0.58	0.25 *p* = 0.30	−0.33 *p* = 0.17	−0.12 *p* = 0.62	0.10 *p* = 0.70
	Maternal coactive onset ratio	Upper-body	0.36 *p* = 0.13	−0.20 *p* = 0.41	0.17 *p* = 0.48	−0.65 *p* = 0.003	0.35 *p* = 0.15	−0.24 *p* = 0.32	−0.06 *p* = 0.81	0.14 *p* = 0.58
		Arms	0.41 *p* = 0.08	−0.16 *p* = 0.50	0.37 *p* = 0.11	−0.29 *p* = 0.22	0.46 *p* = 0.05	−0.27 *p* = 0.27	−0.23 *p* = 0.34	0.30 *p* = 0.22
		Head	0.35 *p* = 0.15	−0.20 *p* = 0.42	0.09 *p* = 0.72	−0.35 *p* = 0.15	0.20 *p* = 0.42	−0.11 *p* = 0.65	−0.14 *p* = 0.56	0.19 *p* = 0.45

*Color indicates that the correlation is statistically significant at the 0.05 level (2-tailed)*.

### Relations between global motion silence and interaction quality

Second, we hypothesized motion silence to be positively correlated with adaptive interaction qualities and negatively correlated with aversive interaction qualities. As shown in Tables 2, 4, this hypothesis was most strongly supported at 4 months, where the silence ratios showed both positive correlations with adaptive interaction qualities and negative correlations with aversive interaction qualities. More specifically, the silence ratios for both upper-body and head motion were positively correlated with child-led, the silence ratios for upper-body and arm motion were positively correlated with maternal sensitivity and negatively correlated with maternal intrusiveness, and the silence ratios for arm motions were further positively correlated with dyadic reciprocity and negatively correlated with parent-led. At 13 months, the silence ratios were solely negatively correlated with the aversive interaction qualities; the silence ratio for head motion was negatively correlated with infant negativity, while the silence ratio for arm motion was negatively correlated with parent-led.

**Table 4:** Spearman's correlations between global motion features and CIB Composites for mother-infant dyads at 13 months (*N* = 33).

**13 m**	**Global motion features**	**Body part**	**CIB Composites**									
			**Maternal sensitivity**	**Maternal intrusiveness**	**Maternal limit–setting**	**Infant involvement**	**Infant negative emotionality**	**Infant compliance**	**Dyadic reciprocity**	**Dyadicnegativestates**	**Parent–led**	**Child–led**
Individual parameters	Infant activity ratio	Upper-body	−0.18 *p* = 0.31	0.31 *p* = 0.08	−0.23 *p* = 0.20	0.02 *p* = 0.92	0.39 *p* = 0.03	−0.20 *p* = 0.26	−0.13 *p* = 0.47	0.24 *p* = 0.18	0.08 *p* = 0.65	−0.05 *p* = 0.79
		Arms	−0.18 *p* = 0.31	0.22 *p* = 0.23	−0.16 *p* = 0.38	0.12 *p* = 0.51	0.25 *p* = 0.16	−0.08 *p* = 0.66	−0.06 *p* = 0.72	0.23 *p* = 0.21	0.05 *p* = 0.77	0.003 *p* = 0.99
		Head	−0.14 *p* = 0.45	0.30 *p* = 0.09	−0.17 *p* = 0.34	−0.02 *p* = 0.90	0.45 *p* = 0.01	−0.19 *p* = 0.28	−0.15 *p* = 0.42	0.22 *p* = 0.22	0.10 *p* = 0.59	−0.06 *p* = 0.73
	Maternal activity ratio	Upper-body	−0.06 *p* = 0.76	0.14 *p* = 0.45	0.06 *p* = 0.78	−0.07 *p* = 0.70	0.30 *p* = 0.09	−0.05 *p* = 0.79	−0.05 *p* = 0.80	−0.07 *p* = 0.72	0.27 *p* = 0.14	−0.27*p* = 0.13
		Arms	−0.19 *p* = 0.28	0.32 *p* = 0.07	−0.06 *p* = 0.75	−0.19 *p* = 0.28	0.31 *p* = 0.08	−0.10 *p* = 0.59	−0.15 *p* = 0.39	−0.06 *p* = 0.74	0.51 *p* = 0.002	−0.47 *p* = 0.01
		Head	0.08 *p* = 0.65	−0.06 *p* = 0.73	0.19 *p* = 0.29	0.09 *p* = 0.60	0.21 *p* = 0.25	0.08 *p* = 0.65	0.05 *p* = 0.76	−0.03 *p* = 0.85	0.01 *p* = 0.94	−0.06 *p* = 0.76
Dyadic parameters	Overlap ratio	Upper-body	−0.04 *p* = 0.82	0.18 *p* = 0.31	−0.06 *p* = 0.73	−0.01 *p* = 0.97	0.41 *p* = 0.02	−0.16 *p* = 0.38	−0.06 *p* = 0.75	0.06 *p* = 0.75	0.13 *p* = 0.46	−0.13 *p* = 0.47
		Arms	−0.19 *p* = 0.29	0.31 *p* = 0.08	−0.15 *p* = 0.40	−0.07 *p* = 0.72	0.37 *p* = 0.03	−0.15 *p* = 0.42	−0.14 *p* = 0.45	0.11 *p* = .54	0.32 *p* = 0.07	−0.26 *p* = 0.14
		Head	0.07 *p* = 0.70	0.05 *p* = 0.78	0.07 *p* = 0.71	0.11 *p* = 0.55	0.37 *p* = 0.04	−0.06 *p* = 0.73	0.03 *p* = 0.87	0.01 *p* = 0.94	−0.01 *p* = 0.96	0.01 *p* = 0.97
	Silence ratio	Upper-body	0.24 *p* = 0.17	−0.28 *p* = 0.12	0.09 *p* = 0.62	0.08 *p* = 0.68	−0.33 *p* = 0.06	0.05 *p* = 0.79	0.16 *p* = 0.39	−0.14 *p* = 0.44	−0.28 *p* = 0.12	0.27 *p* = 0.13
		Arms	0.26 *p* = 0.14	−0.30 *p* = 0.09	0.05 *p* = 0.79	0.04 *p* = 0.82	−0.28 *p* = 0.12	−0.01 *p* = 0.98	0.12 *p* = 0.51	−0.07 *p* = 0.69	–0.35 *p* = 0.04	0.30 *p* = 0.09
		Head	0.14 *p* = 0.43	−0.19 *p* = 0.29	0.06 *p* = 0.75	0.05 *p* = 0.79	–0.41 *p* = 0.02	0.11 *p* = 0.56	0.14 *p* = 0.43	−0.14 *p* = 0.43	−0.17 *p* = 0.36	0.18 *p* = 0.33

*Color indicates that the correlation is statistically significant at the 0.05 level (2-tailed)*.

### Relations between fine-grained motion features and interaction quality

Third, we hypothesized motion coordination to be positively correlated with adaptive interaction qualities and negatively correlated with aversive interaction qualities. As shown in Tables 3, 5, this hypothesis was most strongly supported by the maternal coactive and alternating onset ratios at 4 months, which showed positive correlations with adaptive interaction qualities and negative correlations with aversive interaction qualities. At 4 months, the maternal alternating onset ratios for upper-body and head motion were positively correlated with maternal sensitivity and infant involvement, the maternal alternating onset ratios for head motion were further positively correlated with dyadic reciprocity and negatively correlated with maternal intrusiveness, dyadic negative states, and parent-led. In addition, the maternal coactive onset ratio for arm motion was positively correlated with dyadic reciprocity, while the maternal coactive onset ratio for upper-body motion was negatively correlated with infant negativity. At 13 months, the maternal alternating onset ratio for head motion was solely negatively correlated with aversive interaction qualities, that is, infant negativity and dyadic negative states. The maternal coactive onset ratios did not show any significant correlations to CIB measures at 13 months.

**Table 5:** Spearman's correlations between fine-grained motion features and CIB Composites for mother-infant dyads at 13 months (*N* = 33).

**13 m**	**Fine-grained motion features**	**Body part**	**CIB Composites**									
			**Maternal sensitivity**	**Maternal intrusiveness**	**Maternal limit–setting**	**Infant involvement**	**Infant negative emotionality**	**Infant compliance**	**Dyadic reciprocity**	**Dyadic negative states**	**Parent–led**	**Child–led**
Dyadic parameters	Infant alternating onset ratio	Upper-body	−0.07 *p* = 0.72	0.19 *p* = 0.30	−0.07 *p* = 0.68	−0.26 *p* = 0.14	0.29 *p* = 0.11	−0.23 *p* = 0.20	0.004 *p* = .98	−0.05 *p* = 0.77	0.17 *p* = 0.34	−0.13 *p* = 0.48
		Arms	−0.01 *p* = 0.95	0.12 *p* = 0.52	0.13 *p* = 0.49	−0.08 *p* = 0.67	0.25 *p* = 0.17	−0.16 *p* = 0.37	0.02 *p* = 0.92	−0.10 *p* = 0.58	0.13 *p* = 0.46	−0.16 *p* = 0.36
		Head	−0.05 *p* = 0.77	0.29 *p* = 0.11	−0.28 *p* = 0.12	−0.16 *p* = 0.38	0.39 *p* = 0.03	–0.38 *p* = 0.03	−0.14 *p* = 0.45	0.17 *p* = 0.36	0.05 *p* = 0.78	−0.18 *p* = 0.31
	Maternal alternating onset ratio	Upper-body	0.10 *p* = 0.57	−0.11 *p* = 0.54	0.19 *p* = 0.29	0.17 *p* = 0.35	−0.26 *p* = 0.14	0.09 *p* = 0.62	0.16 *p* = 0.37	−0.27 *p* = 0.13	−0.15 *p* = 0.41	0.23 *p* = 0.19
		Arms	−0.12 *p* = 0.49	−0.05 *p* = 0.79	−0.02 *p* = 0.90	−0.21 *p* = 0.24	−0.02 *p* = 0.92	0.02 *p* = 0.90	−0.14 *p* = 0.42	0.10 *p* = 0.60	0.03 *p* = 0.86	< 0.01 *p* = 1.00
		Head	0.21 *p* = 0.25	−0.16 *p* = 0.37	0.29 *p* = 0.11	0.26 *p* = 0.14	–0.38 *p* = 0.03	0.07 *p* = 0.69	0.28 *p* = 0.11	–0.40 *p* = 0.02	−0.24 *p* = 0.17	0.22 *p* = 0.22
	Infant coactive onset ratio	Upper-body	−0.01 *p* = 0.97	0.27 *p* = 0.13	−0.04 *p* = 0.81	−0.01 *p* = 0.94	0.31 *p* = 0.08	−0.24 *p* = 0.19	−0.16 *p* = 0.38	0.01 *p* = 0.95	0.22 *p* = 0.22	−0.22 *p* = 0.23
		Arms	−0.32 *p* = 0.07	0.27 *p* = 0.12	−0.23 *p* = 0.20	–0.41 *p* = 0.02	0.24 *p* = 0.18	−0.18 *p* = 0.31	–0.41 *p* = 0.02	0.30 *p* = 0.09	0.44 *p* = 0.01	−0.46 *p* = 0.01
		Head	0.08 *p* = 0.66	0.02 *p* = 0.92	0.17 *p* = 0.35	< 0.01 *p* = 0.99	−0.01 *p* = 0.96	0.13 *p* = 0.48	< -0.01 *p* = 0.99	−0.15 *p* = 0.42	0.18 *p* = 0.32	−0.21 *p* = 0.23
	Maternal coactive onset ratio	Upper-body	< 0.01 *p* = 0.98	−0.14 *p* = 0.46	0.01 *p* = 0.97	−0.04 *p* = 0.84	0.12 *p* = 0.51	−0.20 *p* = 0.27	0.05 *p* = 0.80	0.08 *p* = 0.68	−0.31 *p* = 0.08	0.25 *p* = 0.17
		Arms	−0.05 *p* = 0.78	−0.01 *p* = 0.95	−0.08 *p* = 0.65	0.02 *p* = 0.93	0.24 *p* = 0.19	−0.19 *p* = 0.29	0.01 *p* = 0.98	0.09 *p* = 0.62	−0.11 *p* = 0.53	0.08 *p* = 0.66
		Head	−0.04 *p* = 0.81	−0.12 *p* = 0.51	−0.15 *p* = 0.41	−0.01 *p* = 0.94	0.11 *p* = 0.56	−0.22 *p* = 0.22	−0.04 *p* = 0.85	0.12 *p* = 0.50	−0.28 *p* = 0.12	0.27 *p* = 0.13

*Color indicates that the correlation is statistically significant at the 0.05 level (2-tailed)*.

The infant coactive and alternating onset ratios did not show correlations in line with our hypothesis at either 4 or 13 months. At 4 months, the infant alternating onset ratios for upper-body and head motion were positively correlated with child-led, but the infant alternating onset ratio for arm motion was positively correlated with infant negativity. The infant coactive onset ratios did not show any significant correlations to CIB measures at 4 months. At 13 months, the infant alternating and coactive onset ratios showed patterns of correlations opposite to those expected by the hypothesis; the infant alternating onset ratio for head motion was positively correlated with infant negativity and negatively correlated with infant compliance, while the infant coactive onset ratio for arm motion was negatively correlated with infant involvement, dyadic reciprocity, and child-led, and positively correlated with parent-led.

## Discussion

The aim of the current study was to increase our understanding of the role of bodily movements in early mother-infant face-to-face interaction. In line with our hypotheses and previous findings (Hammal et al., 2014, 2015a,b; Leclère et al., 2016), we found that global motion features and, to some extent, fine-grained motion features are systematically related to the quality of the interaction.

### Global motion features and interaction quality

Taken together, our results support the first two study hypotheses, as higher levels of motion activity, i.e., infant, maternal or overlapping motion, are related to more aversive and less adaptive interactions, whereas the opposite is true for higher levels of motion silence. Thus, our results seem to support previous findings (Hammal et al., 2014, 2015a,b; Leclère et al., 2016) showing that higher levels of intra-personal distress, such as infant negativity, and inter-personal distress, such as maternal intrusiveness, are related to excessive motion activity.

Infants' expression of negative affect through increased motor activity is considered a sign of adaptive child development, since they, in this way, clearly communicate their state to their parent. Reacting to distress by reducing the level of activity is considered a sign of maladaptive social withdrawal, which is regarded as a risk factor for aversive child development and a risk marker for parental psychopathology (Guedeney et al., 2013). Thus, it is possible that the relationship between increased motor activity and negative emotionality observed in the present study is indicative of adaptive expressions of negative affect. Further, this relation could be modulated by child or parental psychopathology, as some infants may not express negative emotionality through increased motor activity.

Furthermore, our results underline the importance of motion silence in early mother-infant interactions, which, to our knowledge, has only been studied by Leclère et al. (2016). Together with the results from Leclère et al. (2016), our results show that motion silence is positively related to adaptive mother-infant interactions and negatively related to aversive mother-infant interactions. In our study, motion silence was related to on the one hand increased levels of dyadic reciprocity, maternal sensitivity, and child-led interactions, and on the other hand decreased levels of maternal intrusiveness and infant negative emotionality. Based on the understanding of early parent-infant interaction as a cycle between engagement and disengagement, the relation between pauses and adaptive interaction qualities could be explained by the infant's need for disengagement. Pauses may give the infant time to self-regulate and initiate—and hereby become an active participant in the interaction (Stern, 1974; Bowlby, 1988; Beebe, 2010; Guedeney et al., 2013).

However, our results regarding motion silence should be interpreted with caution. Conceptually, higher levels of motion silence can only be related to more adaptive interactions to a certain degree, as an interaction with no or very little motion activity would be characterized as withdrawn and apathetic (Hammal et al., 2015a). As described in the mid-range model, adaptive mother-infant interactions are characterized by a balance between movement and silence. Interactions with high levels of motion silence may not be characterized by high levels of maternal intrusiveness, but neither are they characterized by high levels of maternal sensitivity or dyadic reciprocity. Interestingly, in contrast to the findings in our study, Leclère et al. (2016) found a relationship between high levels of motion silence and low levels of maternal sensitivity. This discrepancy is most likely due to sample characteristics as half of the mothers in the study by Leclère et al. (2016) showed neglect behavior. Unlike the emotionally neglecting mothers included in the study by Leclère et al. (2016), the non-clinical mothers in the present study may not have demonstrated motion silence to a degree which would disrupt the interaction, whereby the aversive effects of high levels of motion silence are not present in our results.

### Fine-grained motion features and interaction quality

Apart from the global motion features, which provide information on the interaction as a whole, we also analyzed the mothers' and infants' motion at a more fine-grained level, aiming at measuring how the coordination between mother and infant is related to the quality of the interaction. Our results partly support our hypothesis and previous studies on both infant-adult and adult-adult interaction (Ramseyer and Tschacher, 2011, 2014; Hammal et al., 2014) suggesting that higher degrees of coordination are related to more adaptive and less aversive interactions. We found that higher levels of maternal coordination were related to less aversive interaction qualities, such as infant negativity and dyadic negative states, at both 4 and 13 months. That is, in adaptive mother-infant interactions, the mother often moves in relation to the beginning or end of infant movements.

Both the maternal alternating and coactive onset ratios were related to more adaptive interactions at 4 months. However, these two patterns could be related to different types of adaptive interactions with regard to both function and level of arousal. As has been proposed previously, alternating coordination may be representative of pleasant give-and-take interactions, which later develop into adaptive adult conversations. On the other hand, coactive coordination might represent joint heightened moments of arousal and function to facilitate interpersonal bonding (Beebe et al., 1977).

The fine-grained motion features related to whether the infant coordinates his or her movement to maternal movement onset or offset were not in line with our study hypothesis, as they did not correlate with interaction qualities in a unified direction. Our results suggest that when measuring the quality of the early mother-infant interaction, it is more important whether the mother's movements follow the infant's than the other way around. Although both mother and infant are active participants in the mother-infant interaction, the mother is the one with the greater capacity and range of resources, for which reason an adaptive mother-infant interaction arises, when she pauses in order to give the infant time to initiate and she follows the infant's initiatives by coordinating her movements to the infant's (Beebe, 2010).

### Differences between arm and head motion

Inspired by the study by Ramseyer and Tschacher (2014), who found that head-synchrony predicted the global treatment outcome in adult psychotherapy, while upper-body synchrony predicted the session outcome, we wished to explore, whether upper-body, arm, and head movements were involved in expressing different types of interaction qualities.

Examining the global motion features at both 4 and 13 months, arm and head motion do not show any opposing relations to the interaction qualities, thus they appear to be involved in expressing the same types of interaction qualities; higher levels of either head or arm movements are related to more aversive interactions. However, comparing the maternal global and fine-grained motion features, head and arm movements seem to be involved in expressing different interaction qualities. The global level of arm motion, but not head motion, was associated with more aversive interactions at both 4 and 13 months. On the other hand, the level of maternal coordinated head motion, but not arm motion, was associated with less aversive interactions, such as less infant negativity and dyadic negative states, at both 4 and 13 months. This may reflect that the mothers express intrusive behavior through increased arm movements, e.g., waving or clapping, while they express more sensitive behavior through coordinated head movements, e.g., by following the infant's head movements. For the infant, the picture is not as clear; infant negativity is positively correlated with infant arm movements at 4 months but with infant head movements at 13 months.

### Developmental aspects

Given the low number of participants for which data were usable at both 4 and 13 months, it was not possible to statistically compare the correlations in the two groups. However, our results give rise to thoughts on how age may affect the role of movement in mother-infant interaction. Notably, the relations between motion features and interaction qualities seem to be stronger at 4 compared to 13 months. For instance, while we found moderate to strong correlations between both global and fine-grained motion features and maternal intrusiveness and sensitivity at 4 months, we did not find such associations at 13 months. An explanation for this could be that at 4 months mothers more often perform intrusive behavior using physical manipulation or other types of motion activity, such as waving or clapping to redirect the infant's attention, while the mothers at 13 months perform intrusive behavior through the use of other modalities, such as calling the infant's name to redirect his or her attention. Yet, in the study by Leclère et al. (2016), the motion features showed strong correlations to maternal sensitivity and intrusive behavior at infant ages above 12 months. The reason why we could not replicate this finding could be related to differences in sample characteristics, i.e., non-clinical versus clinical sample, or interaction set-up, i.e., unstructured free play versus pre-structured play situation. Taken together, this suggests that the role of motion features in mother-infant interaction may change both in response to infant age and interaction set-up. However, the moderate to strong correlations between motion features and infant negativity are consistent at both 4 and 13 months, demonstrating that the infants consistently express negativity through the motor modality, and that this quality may not change according to infant age.

### Limitations and future research

Our study has some limitations, which need to be acknowledged when interpreting the results. First, due to the low number of mother-infant dyads for which data were present at both 4 and 13 months, we chose not to conduct multivariate analyses and were therefore not able to statistically investigate the effect of age on the relations between motion features and interaction qualities. Second, due to a high amount of missing data, it was not possible to perform analyses of infant leg movements. Finally, the correlational nature of the analyses and the global nature of the rating system used (CIB) only allows conclusions at a global level, and makes it impossible to state the direction of the effects. For instance, we cannot determine whether the periods with heightened levels of overlapping activity are also the periods with heightened levels of child negativity. Likewise, we cannot determine whether the infant becomes negative due to high levels of overlapping motion activity, or whether the high levels of overlapping motion activity are present due to high levels of infant negativity.

Results from the present study suggest that the relationships between motion features and interaction qualities may change across infant age. However, future studies should systematically investigate and clarify how motion features and interaction qualities are related at different infant ages. Further, the mother-infant interactions in the present study were conducted in an observation room, as previous studies have found mothers and infants to interact differently in the laboratory compared to at home (Lewedag et al., 1994; Jaffe et al., 2001), future studies should investigate the relationships between motion features and interaction qualities in home settings. Finally, studies using more fine-grained measures, such as micro-coding (Beebe and Steele, 2013), investigating the sequencing of movement and different interaction qualities, and hereby informing on the direction of effects, are encouraged.

## Conclusion

The present study shows that automatically extracted global and, to some extent, fine-grained motion features are associated with observed interaction qualities in mother-infant face-to-face interactions at infant age 4 and 13 months. Our results demonstrate that higher levels of infant movement are related to more infant negative emotionality at both 4 and 13 months. In addition, at 4 months, higher levels of maternal movement and overlap are related to more maternal intrusiveness, and higher levels of infant movement are related to less maternal sensitivity. On the other hand, higher levels of motion silence are related to more adaptive mother-infant interactions at 4 months, including more maternal sensitivity, dyadic reciprocity, and child-led interactions, i.e., interactions focusing on the infant's needs. Moreover, analyses of fine-grained motion features show that higher levels of maternal coordination are related to less aversive interactions, such as lower levels of dyadic negative states.

Taken together, our results suggest that adaptive mother-infant interactions are characterized by mothers accepting their infants' need for disengagement and social withdrawal by allowing the presence of pauses, and mothers coordinating their movements to their infants' movements. We hypothesize that, in both cases, the infant becomes an active participant in the interaction: during pauses the infant gains time to initiate, and when the mother follows the infant's movements, she accepts that the infant is taking the lead in the interaction.

Finally, we found more and stronger correlations between motion features and interaction qualities at 4 months compared to at 13 months, suggesting that the importance of movement in mother-infant interactions may change according to infant age. However, due to sample size limitations, it was not possible to statistically examine the effect of age on the relationships between motion features and interaction quality. Thus, a venue for future research is to examine, how infant age affects the way movement is related to the quality of parent-infant interactions.

## Ethics statement

This study was carried out in accordance with the recommendations of The Danish Code of Conduct for Research Integrity, Ministry of Higher Education and Science with written informed consent from all subjects. All subjects gave written informed consent in accordance with the Declaration of Helsinki. The protocol was approved by the Research Ethics Committee, Department of Psychology, University of Copenhagen.

## Author contributions

SK, MV, MC, and DC: designed the study; KC and JS-N: completed the CIB coding; IE and GV: completed the analyses; MC and DC: supervised the analyses; IE: wrote the initial draft. All authors interpreted the findings, edited the drafts, and accepted the final version of the article.

### Conflict of interest statement

The authors declare that the research was conducted in the absence of any commercial or financial relationships that could be construed as a potential conflict of interest.

## Footnotes

^1^www.qualisys.com

^4^The time-window of 1.5 s were adopted from previous studies (Delaherche et al., 2013; Leclère et al., 2016).
