# Erratum: Relations between Automatically Extracted Motion Features and the Quality of Mother-Infant Interactions at 4 and 13 Months

**DOI:** 10.3389/fpsyg.2018.00062

**Published:** 2018-01-19

**Authors:** Frontiers Production Office

**Affiliations:** Frontiers Production Office, Lausanne, Switzerland

**Keywords:** movement, motion features, mother-infant interaction, interaction quality, coding interactive behavior

Due to a typesetting error, affiliation 2 was incorrectly assigned to author David Cohen and Mohamed Chetouani. The correct affiliation was missing and should have been “Institut des Systèmes Intelligents et de Robotiques, Centre National de la Recherche 7222, Sorbonne Université Pierre et Marie Curie, Paris, France.”

The publisher apologizes for this error and the original article has been updated to reflect this. This error does not change the scientific conclusions of the article in any way.

